# An open label study to determine the effects of an oral proteolytic enzyme system on whey protein concentrate metabolism in healthy males

**DOI:** 10.1186/1550-2783-5-10

**Published:** 2008-07-24

**Authors:** Julius Oben, Shil C Kothari, Mark L Anderson

**Affiliations:** 1 Laboratory of Nutrition and Nutritional Biochemistry, Department of Biochemistry, University of Yaounde I, Careroon; 2 Gateway Health Alliances, Inc., Fairfield, CA 94534, USA; 3 Triarco Industries, Inc. 400 Hamburg Turnpike, Wayne, NJ 07418, USA

## Abstract

**Background:**

Current research suggests that protein intake of 1.5 – 2.8 g/kg/day (3.5 times the current recommended daily allowance) is effective and safe for individuals trying to increase or maintain lean muscle mass. To achieve these levels of daily protein consumption, supplementing the diet with processed whey protein concentrate (WPC) in liquid form has become a popular choice for many people. Some products have a suggested serving size as high as 50 g of protein. However, due to possible inhibition of endogenous digestive enzymes from over-processing and rapid small intestine transit time, the average amount of liquid WPC that is absorbed may be only 15 g. The combined effect of these factors may contribute to incomplete digestion, thereby limiting the absorption rate of protein before it reaches the ceacum and is eliminated as waste. The purpose of this study was to determine if Aminogen^®^, a patented blend of digestive proteases from *Aspergillus niger *and *Aspergillus oryzae*, would significantly increase the in-vivo absorption rate of processed WPC over control values. It also investigated if any increase would be sufficient to significantly alter nitrogen (N2) balance and C-reactive protein (CRP) levels over control values as further evidence of increased WPC absorption rate.

**Methods:**

Two groups of healthy male subjects were assigned a specified balanced diet before and after each of two legs of the study. Subjects served as their own controls. In the first leg each control group (CG) was dosed with 50 g of WPC following an overnight fast. Nine days later each test group (TG) was dosed following an overnight fast with 50 g of WPC containing either 2.5 g (A2.5) or 5 g (A5) of Aminogen^®^. Blood samples were collected during each leg at 0 hr, 0.5 hr, 1 hr, 2 hr, 3 hr, 3.5 hr and 4 hr for amino acid (AA) and CRP analyses. The following 18 AAs were quantified: alanine, arginine, aspartic acid, cysteine, glutamic acid, glycine, histidine, isoleucine, leucine, lysine, methionine, phenylalanine, proline, serine, threonine, tryptophan, tyrosine and valine. Urine was collected for 24 hours from 0 hr for total N2 analysis. Results are expressed as means ± SEM. All significance and power testing on results was done at a level of alpha = 0.05. Area under the concentration time curve (AUC) was calculated using the trapezoidal rule. One-way analysis of variance (ANOVA-1) was done between CGs, between TGs and between time points. One-way repeated measures analysis of variance (ANOVA-1-RM) was done to compare CGs and TGs. Two-way analysis of variance (ANOVA-2) was performed on total serum amino acid (TSAA) levels, urine N2 levels and CRP levels between each CG and TG.

**Results:**

After baseline subtraction the mean AUC was significantly (p ≤ 0.05) greater in each TG compared the corresponding CG. Comparison of the mean AUC between each TG and each CG was not significantly different. Total serum amino acid (TSAA) levels were significantly greater in each TG compared the corresponding CG. They were also significantly different between each TG but not between each CG. All individual serum amino acid (ISAA) levels in TG-A2.5 except glycine, histidine, methionine and serine were significantly higher than in CG-A2.5 at 4 hr. All ISAA levels in TG-A5 except methionine and serine were significantly higher than in CG-A5 at 4 hr. The N2 balance was significantly higher in each TG compared to the corresponding CG, but not significantly different between each CG and between each TG. Significant differences in CRP levels are reported between each TG compared to the corresponding CG, but not significantly different between each TG and between each CG.

**Conclusion:**

A patented blend of digestive proteases (Aminogen^®^) increased the absorption rate of processed WPC over controls, as measured by statistically significant increases in AUC, TSAA levels, ISAA levels and N2 balance. Significant decreases in CRP levels and fluxes in AA levels are also reported.

## Background

Increased protein consumption has become increasingly popular among individuals, especially athletes, trying to increase or maintain lean muscle mass. Whey protein concentrate (WPC) in liquid form has become a popular protein supplement because it has been characterized as rapidly and easily digestible. Clinical studies have reported that approximately 30 g of WP as a liquid meal produced a large but transient rise in postprandial plasma AA levels in approximately 90 minutes and returned to baseline within 5 hours. The rise in AA levels increased protein synthesis and nitrogen (N2) balance but did not inhibit whole body protein breakdown [1,2]. Recently, it has been reported that the intake of WP above 1.5 g/kg/day helped decrease body fat, increase lean body mass and maintain nitrogen (N2) balance [3]. Protein intake as high as 2.8 g/kg/day (3.5 times the current recommended daily allowance) was reported to have no adverse effects on renal function [4]. To help achieve these high levels of protein intake, some WPC products have a suggested serving size of 50 g. However, several factors could limit protein digestion and absorption at this level of consumption. Digestion may be compromised by industrial-scale WPC production techniques, such as spray-drying and pressurized microfiltration, which have been reported to inhibit the activity of digestive enzymes in-vitro due to over-processing [5]. The addition of proteolytic enzymes to WPC solutions has been reported to increase the degree of hydrolysis (DH), solubility and concomitant in-vitro digestibility (IVD) [6]. Absorption may be compromised by the rapid transit time through the small intestine to the ceacum, which has been reported to be an average of 1.5 hours for viscous liquids [7]. The maximum absorption rate of WP has been reported to be 8 to 10 g/hr [8]. Using these parameters, the maximum amount of WP that could be absorbed from a liquid is 15 g. The combined effects of over-processing and increased intake may contribute to incomplete WPC digestion. This could reduce some of the positive therapeutic effects of a high protein diet including increased lean muscle mass, increased N2 retention and positive cardiovascular effects such as reduced levels of C-reactive protein (CRP) [9-11]. Increasing the amount of WPC digested, before it reaches the ceacum, may increase the absorption rate and the desired outcome of a high protein diet.

Therefore, we hypothesize that the addition of digestive proteases would increase the absorption rate of WPC in-vivo, produce a positive N2 balance and decrease CRP levels. Changes in WPC absorption rate and CRP levels are determined by comparing levels of total amino acids (TSAA), individual amino acids (IAA) and CRP levels in the serum of a control group (CG) to serum levels in a test group (TG). Differences in N2 balance are determined by comparing the total urine N2 excreted in a CG to total urine N2 excreted in a TG.

## Methods

### Experimental design

Two groups of healthy, male subjects were dosed with 50 g of WPC as CGs and nine days later with 50 g of WPC containing either 2.5 g or 5 g of a patented proteolytic enzyme formula from food grade *Aspergillus niger *and *Aspergillus oryzae *as TGs (Aminogen^®^, Triarco Industries, Wayne, NJ). Serum levels of postprandial amino acids (AAs) were used to monitor differences in WPC absorption rate and urine levels of urea were used to determine total N2 [12].

### Whey protein concentrate

The protein used in this study was dry WPC, 85% protein on a dry basis, 6% fat, 3% ash and 6% lactose (Alacen 131, NZP North America). It was recovered from cheese production, processed by membrane separation and spray-dried. Natural vanilla (0.2%) (Craftmaster) was added for flavor. No solubilizers, emulsifiers or other excipients were added. Control and test samples were supplied in pre-measured individual dose packets of 50 g each (42.5 g protein). Control WPC contained no proteolytic enzymes and the test WPC was blended with either 2.5 or 5 g of Aminogen^®^, (US patent # 5,387,422).

### Subjects

Two groups of twenty-one healthy, lean, males ages 19–35, each with a body mass index (BMI) ranging from 20 to 24, volunteered for this study. None of the participants were following any particular protein-rich dietary regime, muscle-toning or bodybuilding program during the study. The study was approved and performed in accordance with the University of Yaounde I, Cameroon Institutional Review Board (IRB). Each participant was informed of the purpose, methods, and possible risks associated with the study, and informed consent was obtained from each participant. All but one participant completed the study and was not included in any statistical analyses.

### Dietary protocol

Participants followed a specified, balanced diet of 2200 Kcal/day during a one week standardization period prior to the start of the study and for nine days between each leg of the study. An overnight fast was required before Day 1 and Day 2 of testing. Participants resumed the diet after the last blood draw on Day 1. Nutrient composition consisted of 40% carbohydrate, 25% protein and 35% fat. The precise weight and composition was established for each individual by a registered dietician at the University of Yaounde Teaching Hospital (CHU). Participants received a main meal of the day at the Laboratory of Nutrition and Nutritional Biochemistry, University of Yaounde 1, and given precise take-out portions for their other meals. Participants were advised to strictly follow their prescribed diets during the study period.

### Control group

Before the study, all participants (n = 41) followed the standardized diet in the dietary protocol for one week and then reported to the Laboratory of Nutrition and Nutritional Biochemistry after an overnight fast for Day 1 of the study. On Day 1, control samples were collected after all participants ingested one 50 g, pre-measured packet of WPC (42.5 g protein) without Aminogen^®^. The entire contents of each individual serving packet were emptied into 0.5 L of distilled water, vigorously shaken and consumed. Blood samples were collected at 0 hr (baseline, immediately prior to ingestion) 0.5 hr, 1 hr, 2 hr, 3 hr, 3.5 hr and 4 hr to measure TSAA, IAA and CRP levels. Urine was collected over 24 hours from each participant at the start of fasting and pooled to measure total N2 excretion. N2 excretion from sweat and feces was not measured. The CGs were designated as CG-A2.5 (n = 21) and CG-A5 (n = 20).

### Test groups

Following a second standardization period of nine days and an over-night fast, the participants returned to the Laboratory of Nutrition and Nutritional Biochemistry for Day 2 of the study. Each participant was randomly assigned to one of two TGs, receiving either 2.5 g Aminogen^®^, pre-blended in 50 g WPC (n = 21) or 5 g of Aminogen^®^, pre-blended in 50 g WPC (n = 20) designated as TG-A2.5 and TG-A5. The entire contents of each individual serving packet containing Aminogen^® ^were emptied into 0.5 L of distilled water, vigorously shaken and consumed. Test data was determined from blood and urine. Blood samples were collected at 0 hr (baseline, immediately prior to ingestion) 0.5 hr, 1 hr, 2 hr, 3 hr, 3.5 hr and 4 hr to measure TSAA, IAA and CRP levels. Urine was collected over 24 hours and pooled to measure total nitrogen excretion. N2 excretion from sweat and feces was not measured. Following the study, the control data from each participant was compared to the data from the corresponding patient in either of the two test groups.

### Sample collection

Whole blood samples (approximately 5 mL) were collected by a phlebotomist from either an affixed catheter or multiple venous punctures, and transferred to plain Vacutainer^® ^tubes. Serum was prepared by centrifugation and stored in 200 μl aliquots at -20°C until needed for analyses. Prior to each blood draw at 0 hr, 2 hr and 4 hr, heart rate (HR) and blood pressure (BP) were recorded by an attending technician. Pooled urine from each patient in each CG and TG was collected and refrigerated until analyzed for nitrogen.

### Analytical analyses

All serum and urine samples were submitted to the laboratory blinded to remove any analytical bias.

Amino acid (AA) analyses consisted of quantification of eighteen individual serum amino acids for each patient at each time point. Analyses were performed on a Beckman 6300 AA analyzer using ion exchange chromatography and a post column derivatazation with ninhydrin and UV detection. Quantification was done versus reference standard mixtures and control mixtures of known quantities of all eighteen AA (Sigma, St. Louis, MO). Within run variations in control values of greater than +/- 20% required reanalysis. AA levels are reported in mg/L and as percent AUC. TSAA levels were reported as the total sum of all eighteen AAs. The percent AUC was reported as the amount each AA contributed to the total. Urinary nitrogen was determined manually by the AOAC Kjeldahl digestion flask method [13]. Within run variations in control values of greater than +/- 20% required reanalysis. CRP was measured by a highly sensitive immunoturbidimetric assay (Dade Behring, Paris France) by forming an immune complex with specific antibodies. Within run variations in control values of greater than +/- 20% required reanalysis.

### Statistical analyses

All significance and power testing on results was done at a level of alpha = 0.05. Within group analyses was done between each corresponding CG and TG and between group analyses was done between both CGs and between both TGs. The mean area under the concentration time curve (AUC) was calculated after baseline subtraction using the trapezoidal rule. One-way repeated measures analysis of variance (ANOVA-1-RM) was performed on mean AUC between each corresponding CG and TG. One-way analysis of variance (ANOVA-1) was performed on mean AUC between both CGs and between both TGs. Two-way analysis of variance (ANOVA-2) was performed on TSAA levels between each corresponding CG and TG. ANOVA-1 was performed on TSAA levels between 0 hr and each time point within each CG and TG and on area percents of each IAA level at 0 hr and 4 hr. ANOVA-2 was used on the urine N2 and CRP levels between each corresponding CG and TG. Results are expressed as means ± SEM. Statistics were performed using a commercially available software program (Origin^® ^for Windows, version 8.0).

## Results

### Average TSAA levels and AUC

After baseline subtraction, the AUC of TG-A2.5 was 2.2 times greater than the corresponding CG-A2.5 and the AUC of TG-A5 was 3.5 times greater than the corresponding CG-A5. The mean area differences were significantly greater in each TG compared to the corresponding CG (p = 0.04). Mean area differences between both TGs and both CGs were not significantly different.

Analysis of TSAA kinetic profiles of all groups showed a progressive, time-dependant increase through 4 hr. ANOVA-2 comparison of means between CG-A2.5 and the corresponding TG-A2.5 showed differences between groups and all time points to be significant (p = 0.05). The interaction between time points and dose groups was also significant (p = 0.05). A statistical power of 1.0 was reached between all comparisons. Comparison of means between CG-A5 and the corresponding TG-A5 showed differences between groups and all time points to be significant (p = 0.05). The interaction between time points and dose groups was also significant (p = 0.05). A statistical power of 1.0 was reached between all comparisons. Comparison of means between both CGs showed no significant difference between groups, a significant difference between time points (p = 0.05) and no significant interaction between groups. Comparison of means between both TGs showed a significant difference between groups and time points (p = 0.05) but no significant interaction between groups.

**Figure 1:**
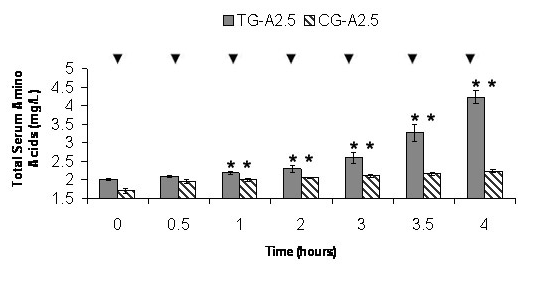
**CG-A2.5 and TG-A2.5 Average Total Serum Amino Acids (TSAA)**. Average levels of TSAA in Control Group (CG) A2.5 (n = 21) and Test Group (TG) A2.5 (n = 21). Values are means ± SEM. * Indicates significant change from baseline (p = 0.05). ▼ Indicates significant difference between groups (p = 0.05).

**Figure 2:**
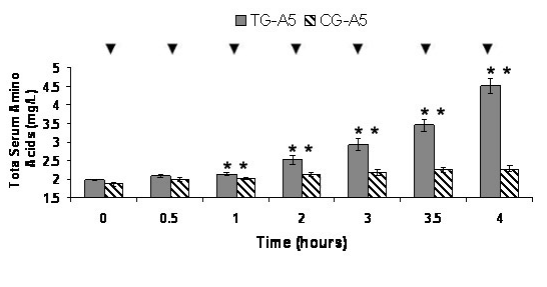
**CG-A5 and TG-A5 Average Total Serum Amino Acids (TSAA)**. Average levels of TSAA in Control Group (CG) A5 (n = 21) and Test Group (TG) A5 (n = 21). Values are means ± SEM. * Indicates significant change from baseline (p = 0.05). ▼ Indicates significant difference between groups (p = 0.05).

ANOVA-1 comparison of mean TSAA levels between 0 hr and each time point showed significant (p ≤ 0.05) increases in all TGs and CGs. In CG-A2.5, the mean 0 hr TSAA level of 1.71 mg/L increased significantly at each time point, except the 0.5 hr to a maximum level of 2.22 mg/L in 4 hr. In TG-A2.5 the mean 0 hr TSAA level of 2.01 mg/L increased significantly at each time point except the 0.5 hr to a maximum level of 4.23 mg/L in 4 hr (Figure 1). In CG-A5, the mean 0 hr TSAA level of 1.87 mg/L increased significantly at each time point, except the 0.5 hr to a maximum level of 2.28 mg/L in 4 hr. In TG-A5 the mean 0 hr TSAA level of 1.99 mg/L increased significantly at each time point except the 0.5 hr to a maximum level of 4.52 mg/L in 4 hr (Figure 2).

### Individual amino acid levels

**Figure 3:**
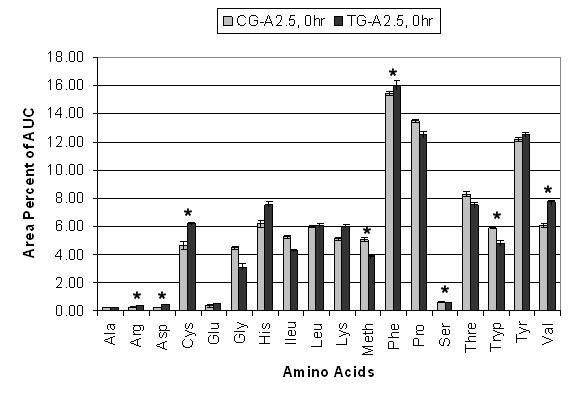
**Individual Amino Acid AUC 0 hr**. Average percent area under the curve (AUC) differences between eighteen amino acids in CG-A2.5 (n = 21) and TG-A2.5 (n = 21) at 0 hr. Values are means ± SEM. * Indicates significant difference (p = 0.05).

**Figure 4:**
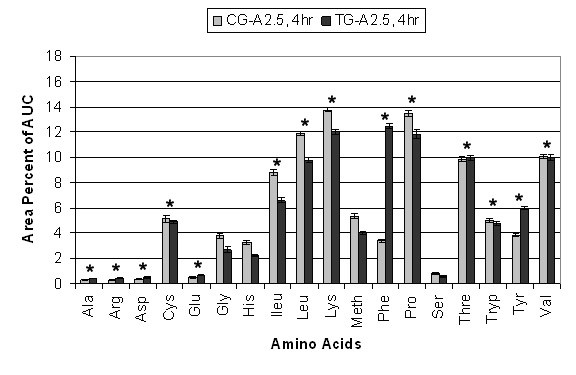
**Individual Amino Acid AUC 4 hr**. Average percent area under the curve (AUC) differences between eighteen amino acids in CG-A2.5 (n = 21) and TG-A2.5 (n = 21) at 4 hr. Values are means ± SEM. * Indicates significant difference (p = 0.05).

The relative percent AUC for each of the eighteen AAs analyzed in CG-A2.5 and TG-A2.5 at 0 hr and 4 hr is shown in Figure 3 and 4. The sum of the average area percent for each AA equals 100% of the AUC. ANOVA-1 statistical analysis between CG-A2.5 and TG-A2.5 at 0 hr showed arginine, aspartic acid, cysteine, methionine, phenylalanine, serine, tryptophan and valine (8 of 18) to be significantly (p ≤ 0.05) different. No significant differences were found between alanine, glutamine, glycine, histidine, isoleucine, leucine, lysine, proline, threonine and tyrosine. At 0 hr statistically significant differences between CG-A5 and TG-A5 include arginine, aspartic acid, glycine, histidine, isoleucine, lysine, methionine, proline, serine and threonine (10 of 18). No significant differences were found between alanine, glutamine, cysteine, leucine, phenylalanine, tryptophan, tyrosine and valine. At 4 hr statistically significant differences between CG-A2.5 and TG-A2.5 include alanine, arginine, aspartic acid, cysteine, glutamine, isoleucine, leucine, lysine, phenylalanine, proline, threonine, tryptophan, tyrosine and valine (14 of 18). No significant differences were found in glycine, histidine, methionine and serine. At 4 hr statistically significant differences between CG-A5 and TG-A5 include alanine, arginine, aspartic acid, cysteine, glutamine, glycine, histidine, isoleucine, leucine, lysine, phenylalanine, proline, threonine, tryptophan, tyrosine and valine (16 of 18). Methionine and serine were the only two amino acids that were not significantly different.

Some AAs, including lysine, phenylalanine, tyrosine and the branched chain amino acids (BCAAs) isoleucine, leucine and valine, showed significantly greater fluctuation between 0 hr to 4 hr in each CG compared to each TG. CG-A2.5 showed a 78% decrease in the AUC of phenylalanine and a 69% decrease in tyrosine, compared to a 22% decrease and a 52% decrease, respectively, in TG-A2.5. These decreases are compensated for by a 170% increase in the AUC of lysine and a 232% increase in the AUC of BCAAs in CG-A2.5, compared to a 100% increase and a 144% increase respectively TG-A2.5.

CG-A5 showed a 74% decrease in the AUC of phenylalanine and a 66% decrease in tyrosine between 0 hr and 4 hr, compared to an 18% decrease and a 72% decrease, respectively, in TG-A5. These decreases are compensated for by a 120% increase in the AUC of lysine and a 259% increase in the AUC of BCAAs in CG-A5 compared to an 85% increase and a 163% increase respectively TG-A5.

### Nitrogen excretion

**Figure 5:**
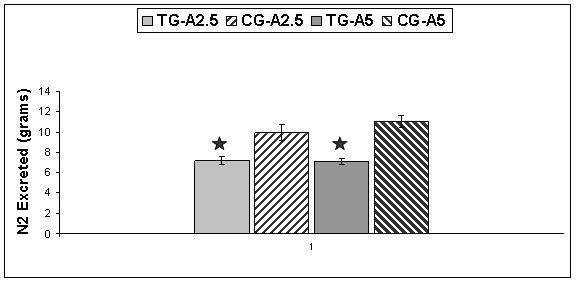
**Total Nitrogen Excretion**. Average 24 hour nitrogen excretion between Test Group (TG) A2.5 and Control Group (CG) A2.5 (n = 18). Average 24 hour nitrogen excretion between TG-A5 and CG-A5 (n = 19). Values are means ± SEM. * Indicates significant difference between corresponding TG and CG (p = 0.05).

The average amount of N2 excreted in 24 hours was determined as urea for each TG and CG. ANOVA-2 showed that the mean N2 excretion decreased significantly (p < 0.05) in each TG compared to each corresponding CG. The mean difference between both TGs and between both CGs, as well as the interaction between both TGs and between both CGs is not significant (Figure 5). A statistical power of 1 was reached between each TG and corresponding CG. In TG-A2.5 and TG-A5 the average amount of N2 excreted over 24 hours was 7.18 g and 7.1 g respectively. In CG-A2.5 and CG-A5 the average amount of N2 excreted over 24 hours was 10.02 g and 11.05 g respectively.

### C-reactive protein

**Figure 6:**
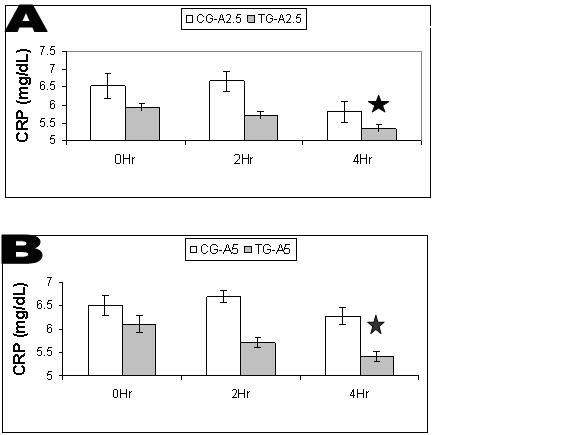
**A. CRP Levels in Control Group (CG) and Test Group (TG) A2.5**. Average C-reactive protein (CRP) levels between CG-A2.5 and TG-A2.5 (n = 16). Values are means ± SEM. * Indicates significant difference (p = 0.05). B. CRP Levels in Control Group (CG) and Test Group (TG) A5. Average C-reactive protein (CRP) levels between CG-A5 and TG-A5 (n = 16). Values are means ± SEM. * Indicates significant difference (p = 0.05).

CRP was measured at 0 hr, 2 hr and 4 hr in each CG and TG (Figure 6). ANOVA-2 comparison of means between each CG and corresponding TG showed significant differences (p = 0.05) between time points and dose groups. A statistical power of 1.0 was reached between each TG and corresponding CG. No significant differences were found when comparing the means of both CGs and both TGs. The interaction between time points was also not significant. No significant changes were found between 0 hr and 2 hr in either CG or TG. Significant (p ≤ 0.05) reductions were found between 0 hr and 4 hr in each TG and not in either CG. TG-A2.5 showed a reduction of 10.12% and TG-A5 showed a reduction of 11.4%.

## Discussion

The purpose of this study was to determine if Aminogen^®^, a patented blend of digestive proteases from *Aspergillus niger *and *Aspergillus oryzae*, would significantly affect the amount of processed WPC metabolized in-vivo and whether any effect would be sufficient to significantly alter N2 balance and CRP levels. Comparing levels of TSAA after ingestion, with and without Aminogen^®^, would indicate if the proteolytic enzymes were effective in increasing the amount of WPC metabolized.

The results show that postprandial TSAA levels were significantly increased over controls from a 50 g dose of Alacen 131 WPC containing either 2.5 or 5 g of Aminogen^® ^(Figures 1 and 2). This indicates that protease supplementation increased the absorption rate amount of the WPC and is further supported by significant (2.2 and 3.5 times) increases in the AUC in each TG relative to each CG. After base line subtraction, no significant differences were found between the AUC of each TG indicating that the WPC absorption rate may close to maximum in TG-A2.5. Further studies may confirm this by increasing the number of TGs consuming various amounts of Aminogen^®^. The significantly higher levels of AAs in the TGs may be related to significant increases in N2 retention and significant decreases in CRP levels. TSAA levels in each TG appeared to be sustained much longer in this study than in earlier studies using a 30 g dose of WP isolate from milk [1,2]. Peak postprandial plasma AA levels in those studies were reported at approximately 1.5 hours and returning to baseline values in approximately five hours. In this study, postprandial TSAA levels appear to have been still increasing or peaked at four hours. Several factors may be responsible for these differences, including the amount ingested and over-processing.

The amount of WPC ingested may have saturated endogenous proteolytic enzymes, the rate-limiting step in the digestion process. Decreased digestion from over-processing can also slow digestive enzymes [5]. These factors impede the absorption rate, which may limit the amount of protein absorbed before reaching the ceacum. The addition of Aminogen^® ^provided more proteolytic enzymes thereby possibly increasing the absorption rate during the same time period. Whether the addition of Aminogen^® ^affected one or more of these factors in this study is unknown at this time but the over-all effect appears to be a significant increase in the WPC absorption rate.

Results of IAA indicate that Aminogen^® ^supplementation may contribute to decreased whole body protein metabolism. Decreased flux of IAA levels such as phenylalanine and valine have been reported to be indicators of decreased whole body protein metabolism and decreased N2 excretion [1,14]. Fluctuations in tyrosine, phenylalanine and BCAAs were compared using average percent AUC. In this model the sum of all eighteen AAs at each time point is equal to 100% of the AUC. Fluctuation in the level of one amino acid must be compensated for by an opposing relative fluctuation in one or more other amino acids. The results show significantly less fluctuation in these essential AAs between 0 hr to 4 hr in each TG than in each CG, indicating decreased whole body protein metabolism. These results, together with significant increases in TSAAs, indicate that Aminogen^® ^supplementation may contribute to optimal conditions for protein synthesis and growth. This is consistent with a significant decrease in average N2 excretion in the TGs of 7.3 g compared to an average of 10.18 g in the CGs (equivalent to approximately 44.6 g and 63.6 g of protein, respectively). These differences offer further support that whole body protein metabolism was decreased in each TG. Further studies can verify this by subtracting baseline urinary N2 levels and measuring N2 excretion each hour during the course of the study and several hours post study. These results also suggest increased protein utilization; however blood urea nitrogen (BUN) and insulin levels should have been monitored to be more certain.

With respect to CRP levels, these results show no significant effect in each CG. However, CRP levels decreased significantly between 0 hr and 4 hr in each TG, A2.5 (p = 0.0038) and A5 (p = 0.0026). This may be related to the differences in the amount of WPC digested and absorbed between each CG and TG. Previous studies have shown that peptides produced from in-vitro, enzymatic hydrolysis of WP are bioactive in reducing CRP [11]. Supplementing with Aminogen^® ^may contribute to significantly reducing CRP levels by producing bioactive peptides in-vivo.

## Conclusion

Factors such as high protein intake and over-processing may contribute to a decreased absorption rate of WPC and lower than expected blood levels of individual amino acids. This may also cause digestion to take longer and be less complete than expected. The results of this study indicate that supplementing 50 g of WPC with 50 mg/g or 100 mg/g Aminogen^®^, a blend of food grade proteases from *Aspergillus niger *and *Aspergillus oryzae *patented for use as a digestive aid, significantly increased the absorption rate of WPC over controls. This is reflected in statistically significant (p ≤ 0.05) increases in postprandial AUC, TSAA levels, ISAA levels and N2 balance. Significant decreases in CRP levels and fluxes in AA levels (possibly contributing to a decrease in whole body protein metabolism) are also reported. Further, these results indicate the need for further research in the area of enzyme supplementation for a more complete digestion of protein and perhaps other processed foods.

## Competing interests

This study was funded by Triarco Industries, Inc. (Wayne, NJ) through the contract research organization (CRO) Gateway Health Alliances, Inc. All research was conducted independently and according to protocol at the Laboratory of Nutrition and Nutritional Biochemistry, University of Yaounde I, Cameroon. None of the researchers have any financial interests concerning the outcome of this investigation and the results do not constitute an endorsement by the authors and/or their institutions concerning the ingredient tested.

## Authors' contributions

JO assisted in study coordination, supervision and data collection. SCK assisted in protocol development, data management and statistical analysis. MLA assisted in protocol development, clinical supply management and manuscript preparation. All authors read and approved the final manuscript.
